# Obstetrics and Gynecology

**Published:** 1877-04

**Authors:** 


### I. Obstetrics and Gynecology.

*Puerperal Fever and Septicæmia.* George Hunter, Scotland. (*Edinburgh Med. Jour.*, 1876.)

Dr. Hunter's aim is to furnish conclusive clinical evidence that puerperal fever may produce septicæmia, and that septicæmia may produce what has been termed puerperal fever. It is with reluctance that we refrain from reproducing the whole article under the head of "Selections," in another department of The Journal and Examiner. Several cases, *ad rem*, are reported by H., which indicate unmistakably the position he defends:

The first case affected with puerperal fever was that of Mrs. A., the mother of two healthy children, who aborted on the 23d February, 1876, at the third month.

The hemorrhage was not by any means profuse, but the decidua required some rather tedious manipulation for their complete removal; and it was noticed that portions of them had an offensive odor. On the third day after parting with the uterine contents, she had a rigor, followed by severe pain over the uterine region, with nausea, frequent weak pulse, increased temperature (103°), furred tongue, and marked diminution of the lochial discharge.

From this time until March 3d, when she died, the pulse became more frequent, ranging from 120 to 140, weak and thready; the tongue sodden and bile-stained, but not altogether dry; vomiting, principally of bilious, but latterly of most offensive matter, with a slight stercoraceous fetor, severe and sustained; expression anxious, but mental faculties preserving their usual alertness; the abdomen increased in distension, with the intestinal convolutions distinct and flatus retained, until respiration became shallow and embarrassed; and then the fall of temperature, with pinched expression, coldness of the breath, forehead, and nose, and icy feeling of the extremities ushered in the fatal termination.

Mrs. Z. having been engaged to be with Mrs. A. at her confinement, came to her as nurse when she aborted, and slept in the same room, on a bed quite close to, and almost alongside, her patient. Mrs. Z. was instructed and directed to make frequent vaginal injections and vulvar ablutions, which she carried out very carefully and satisfactorily, both with Condy's fluid and carbolic-acid lotion. She was almost in constant attendance on Mrs. A. during the night, and was seldom able to undress completely for the few hours she might be able to snatch, while her patient was being watched by some of her own relatives.

Four or five days after Mrs. A.'s funeral (11th or 12th March) Mrs. Z.

went home to her own family, which consisted of her husband, a man about 66 years of age, and an imbecile son of 18 years.

Ten or twelve days after her return, her husband exhibited symptoms of virulent blood-poisoning. These were violent rigors, followed by fever, thirst, frequent weak pulse, which soon became irregular; profuse perspirations, dry brown tongue, delirium, diarrhœa, etc.

A swelling began to form in the right axilla, which did not appear to be acutely painful, and which gradually increased to the size of a melon or cocoa-nut, at the time of his death. Some of the contents were removed by the aspirator and found to contain sanious purulent matter, serum, and tissue *debris*. The urine did not contain albumen, and there was no disease elsewhere.

"The *third* case (Mrs. G.) was attended by my assistant, Dr. Brown, on the 7th March, in her fourth confinement. Labor was easy and natural, and nothing untoward happened until the evening of the second day after delivery, when she complained of coldness of feet and shivering feelings passing up the legs and across the small of the back. Early on the morning of the third day she was seized with severe pain in the left ilio-hypogastric region, rigors, great frequency of pulse, vomiting, and other symptoms, which, with the flabby mammæ, and arrested or greatly diminished discharge, left no doubt in my mind respecting the dangerous, and I may say hopeless, malady with which I had to do. Death took place on the 13th March, six days after delivery, and after only four days' continuance of the more serious symptoms.

The effects to those in attendance upon Mrs. G. were most serious, and demand attention.

Mrs. X., the mother of this patient, who acted as nurse, making frequent vulvar and vaginal cleansings and injections, was seized on the fourth day after her daughter's delivery with prolonged shivering, followed by fever, sickness, great thirst, quick pulse, and acute pain from the right fore-arm to the shoulder. On the following day the right shoulder was swollen, and the supra-clavicular, subclavicular, and axillary glands were enlarged and painful. This was succeeded by alarming constitutional symptoms, with great and severe prostration. The pulse rose to 120, and was frequently irregular, the tongue dry and brown, and there was much thirst, with great mental depression and apathy. To the lymphangitis there were superadded cold shiverings (more than once repeated) and perspirations, and ultimately suppuration of the affected glands ensued. After evacuation of the large abscess which formed at the outer border of the pectoralis major, the symptoms abated, and on the 4th May she was convalescent and gaining strength rapidly. The cicatrix of a slight injury to the dorsal surface of the right index finger, which had escaped observation at the time of its infliction, was now discovered, and Mrs. X. remembered her family calling her attention to it on her return to her own house immediately after her daughter's decease. It then resembled the small blister resulting from a burn, and was filled with white matter. The resulting cicatrix was the size of a shilling.

When Mrs. X. became ill, another daughter attended Mrs. G., and performed the duties of nurse until the latter's death. Three or four days afterwards, when in town shopping, Miss X. had a rigor, accompanied with a feeling of general illness and sharp pain on the dorsal aspect of the joint between the second and third phalanges of the right ring-finger. To much constitutional disturbance succeeded increasing pain, swelling and tension of the dorsal and palmar aspects of the hand, and brawny infiltration of the fore-arm, with red lines indicating the course of the inflamed lymphatics. The local symptoms increased still more in severity, until there were uniform redness, swelling, and tension of the hand, of the fore-arm, and part of the arm, with painfully enlarged glands in the axilla. Free incisions were made over the dorsal and palmar aspects of the ring-finger and hand, which gave exit to a considerable quantity of serum and sanious pus. The lips of the wound remained gaping, in this respect resembling an incision into a piece of liver, and exactly like the incisions into the hand of Mr. Y., to whose case I have already alluded. Miss X. was now removed from the house of her sister in the country to town, when the greatest care was observed in dressing the hand antiseptically morning and evening; and, fortunately, the finger was saved, although amputation at the metacarpo-phalangeal joint, or even of part of the hand, appeared at one time to be unavoidable. Recovery was tedious, there being much vomiting, anorexia, and despondency; but on the 4th June the wounds were completely healed, some stiffness and contraction of the ring-finger only remaining.

One of the servant-maids, who had washed some of the body-linen, soiled by discharges from the deceased, next became affected. She shivered and complained of headache and feverishness, and suffered from sloughy ulceration of the tonsils, with enlargement of the submaxillary glands. She was under the necessity of leaving her situation to go to her own home, and she thus passed from my observation.

Finally, Mr. G., a healthy young farmer, much out in the open air of his upland farm, was affected with general *malaise*, ending in inflamed tonsils, which, however, terminated in resolution after a short illness."

From March 9th to April 1st, H. discontinued obstetric practice, and went into the country, exposed himself freely to the purifying influences of wind and weather. Before returning he took a Turkish bath, made a complete change of clothing, and used the nail brush more diligently, if possible, than before. On the day of his return he was called to three lying-in cases. The first two cases he reached late, and his services were scarcely needed; they made good recoveries. Let the Doctor give his own account of the third case:

"The third case, my *fourth* fatal one from puerperal fever, was that of Mrs. R., a healthy young primipara.

I used carbolic-acid for the fingers of my right hand, and took the precaution to change my coat on my arrival, having borrowed one from the husband for the occasion. In spite of all these precautions, Mrs. R. shivered on the third day, complained of pain over the uterus, and had a slight tendency to diarrhœa.

The course of her case was in every respect similar to the others narrated; but the symptoms were not quite so acute, for death did not occur until 10th April. After the third day, thinking it better and safer for my other patients, I discontinued my visits, and Dr. Baird, my former partner, very kindly undertook the care of this case.

Mrs. X., who lived near at hand, came to be with her daughter at her confinement. She was constantly in the same room with Mrs. R., and, when the latter became seriously ill, slept in the same bed. On the second day (4th April) Mrs. X., who had been very anxious about her daughter, pricked the middle finger of the left hand with a pin when removing the binder, and on the third day became suddenly ill, with shivering, vomiting, and headache. At the time of my visit, she had a pinched, earthy look, with hollow eyes, frequent pulse, and a tendency to retching and giddiness on walking. She was at once removed to her own house, and when seen the following day, she complained of pain in the left shoulder and axilla. On examination, it was found that the axillary and subclavicular glands were enlarged and painful to the touch, and that the arm and fore-arm were swollen and inflamed. Soothing lotions reduced the latter; but the glandular affection increased in severity, and, on the 11th May, a breakfast-cupful of purulent matter was evacuated from an opening at the anterior fold of the axilla, and a considerable amount also from a second opening on the inner aspect of the arm, a couple of inches above the elbow. These two openings communicated, and, on the 8th June, a drainage-tube was introduced.

During the maturation of the suppuration in and around these glands, Mrs. X. was totally unable to leave her bed, and was subject to chills, followed by flushings of heat, generally ending in perspirations, which caused much adynamia. Since the introduction of the drainage-tube, and the more complete evacuation of the offensive discharge, appetite and sleep have returned, and she is now (28th June) convalescent.

A sister-in-law, who attended to Mrs. R. and the infant after Mrs. X.'s illness, and who slept in the same bed on the nights of 6th, 7th, and 8th of April, began at that time to complain of stiffness of one side of the neck when supporting Mrs. R. in bed, and, on the 10th or 11th (April), it was observed that the submaxillary glands of that side were much swollen and painful. An examination of the fauces made the nature of the case apparent; for it was then observed that there was ulceration of the tonsil, with the formation of white sloughing patches on it. Extensive suppuration of the affected glands ensued, and, on the 5th May, the abscess was freely incised, giving vent to a large quantity of healthy pus. She has not yet recovered her strength, and (28th June) still looks uery anæmic.

A *second* sister-in-law assisted at the washing and dressing of the body of Mrs. R., and, in doing so, carelessly put one of the pins into her mouth. On the following day she assisted in washing some of the clothing of the deceased. The same evening she returned to her own home in a neighboring town, and found that her throat was affected. She was confined to the house for some time, under the care of the local medical attendant.

Soon after the infant's birth (9th April), those nursing it observed an offensive discharge from the roots of the finger-nails of both hands. Care was taken to prevent the child putting its fingers into its mouth, and a carbolic-acid lotion having been applied, it soon made a good recovery.

A *third* sister-in-law became affected soon after the opening of the abscess in her sister's neck, with swollen, inflamed, and slightly ulcerated tonsils.

Next, the husband of Mrs. R. was seized eight days after the preceding with acute tonsillitis, which, however, did not go on to suppuration, but ended in resolution.

Finally, the husband of Mrs. X., who had washed some of the bandages and rags soiled by the discharges from his wife's arm, was seized with erysipelatous redness and swelling of his right hand, near the root of the thumb, and between it and the index finger. Under the use of local discutient and sedative lotions, it gradually disappeared without leaving any bad results.

Until quite recently, it was almost universally admitted that puerperal fever was a fever *sui generis*. To-day, however, the most distinguished obstetric authorities have abandoned this theory. Dr. Hunter's cases point to the probable identity of puerperal fever with septicæmia.

